# Implementing an artificial intelligence system into a diabetic eye screening programme in Tanzania

**DOI:** 10.1093/trstmh/trae132

**Published:** 2024-12-16

**Authors:** Charles R Cleland, William U Makupa, Bernadetha R Shilio, Justus Rwiza, David Macleod, Covadonga Bascaran, Matthew J Burton

**Affiliations:** International Centre for Eye Health, London School of Hygiene and Tropical Medicine, London WC1E 7HT, UK; Eye Department, Kilimanjaro Christian Medical Centre, Moshi, P. O. Box 3010, Tanzania; Eye Department, Kilimanjaro Christian Medical Centre, Moshi, P. O. Box 3010, Tanzania; Ministry of Health, Community Development, Gender, Elderly and Children, Dodoma, P. O. Box 743, Tanzania; Eye Department, Kilimanjaro Christian Medical Centre, Moshi, P. O. Box 3010, Tanzania; International Centre for Eye Health, London School of Hygiene and Tropical Medicine, London WC1E 7HT, UK; International Centre for Eye Health, London School of Hygiene and Tropical Medicine, London WC1E 7HT, UK; International Centre for Eye Health, London School of Hygiene and Tropical Medicine, London WC1E 7HT, UK

**Keywords:** artificial intelligence, diabetic retinopathy, implementation, screening

## Abstract

Tanzania has the highest age-adjusted prevalence of diabetes in sub-Saharan Africa. Diabetic retinopathy, a common complication, is a significant cause of vision loss; but with effective screening and treatment this often can be prevented. However, with very few specialist eye care staff in Tanzania this is a major challenge. Artificial intelligence (AI) systems, which automate clinical decision making and therefore task-shift away from specialist staff, could contribute to improved diabetic retinopathy screening services in low-resource settings. This article describes our experiences of selecting, procuring and implementing an AI system into a regional diabetic eye screening programme in northern Tanzania.

## Ophthalmology, artificial intelligence and diabetic eye disease

Ophthalmology, as a heavily image-dependent specialty, is particularly well placed to benefit from advances in healthcare artificial intelligence (AI). Indeed, the first ever autonomous AI-based medical device to receive approval from the United States Food and Drug Administration (FDA) was for diabetic retinopathy (DR) screening^1^ and there are now multiple systems that have received regulatory approval and are therefore licenced to be used as medical devices within diabetic eye screening services.

DR, a common complication of diabetes, is a leading cause of blindness globally, with annual screening for DR recommended by the WHO.^2^ Screening for DR involves capturing retinal photographs from people living with diabetes that are then reviewed by trained personnel for the level of DR. When the level of DR meets the referral threshold, people are advised to attend ophthalmology services for assessment and treatment as required. As DR is generally asymptomatic in the early stages, in the absence of screening, people typically present to eye care services late with advanced disease that is much less responsive to treatment.

Tanzania has the highest age-adjusted prevalence of diabetes in Africa^3^ and, in response to this, has established five diabetic eye screening and treatment programmes across the country. However, with only 0.8 ophthalmologists per million population (against a global average of 31.7),^4^ human resource constraints have limited the impact of these screening programmes. Current screening programmes rely on either ophthalmologists or trained graders to review retinal photographs and because there are so few of these it can mean:

1. The reach of the programmes is limited, with some areas receiving no, or only intermittent, DR screening.
2. When individuals are screened, the retinal photographs are not reviewed in real time and so the results are given to patients a few weeks after screening. This has contributed to low rates of clinic attendance (approximately 40%) for those identified as having potentially sight-threatening DR in need of further management.

The use of AI for DR screening can address both of these challenges. First, by task-shifting away from clinicians, workforce pressures are reduced. Second, by providing a point-of-screening result the patients can be counselled immediately, with evidence suggesting this can significantly improve follow-up rates.

**Figure 1.**
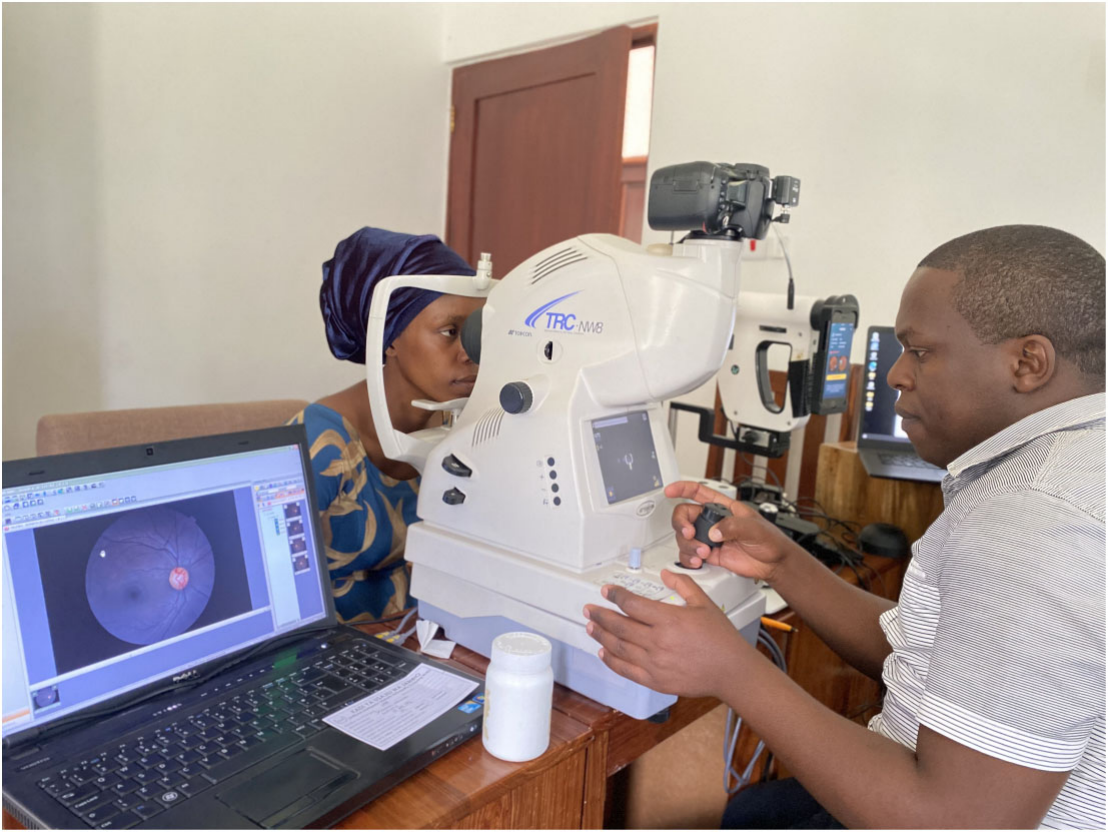
A patient being screened for diabetic retinopathy using artificial intelligence.

In 2023, we implemented an AI system into the Kilimanjaro diabetic eye screening programme as part of a randomised controlled trial, to evaluate the potential role of AI for DR screening in Tanzania, in collaboration with the Tanzanian Ministry of Health and the Kilimanjaro Christian Medical Centre Eye Department.^5^ This AI-assisted DR screening trial has now been running for >12 mo (Figure 1).

## Preimplementation considerations

Prior to implementation it was necessary to decide which of the multiple AI systems that have been developed for DR screening would be most appropriate for a Tanzanian context. As these AI tools are medical devices, and with a view to potentially expanding the implementation of AI screening for DR across Tanzania, we only considered AI systems that had received appropriate regulatory approval. As Tanzania does not currently have a regulatory framework to approve the use of AI-assisted devices, we considered products that had European (CE) or FDA approval as potentially suitable. Some regulatory approvals specify which retinal cameras their product is licenced to be used with. The camera used in the Kilimanjaro programme is a Topcon NW8 camera and therefore any AI system we considered had to be licenced for use with this camera.

Google Health previously implemented their AI software for DR screening into a national screening programme in Thailand and the requirement for their AI system to have an internet connection was noted as a problem and resulted in suboptimal performance. Aware of the limited internet access in Tanzania, particularly in rural district hospitals where the Kilimanjaro DR screening programme operates, another key preimplementation criterion was the ability of the selected AI software to function offline.

Moreover, Tanzania's data privacy laws do not allow the transfer of clinical or imaging data outside of the country without a formal data transfer agreement. The majority of AI systems host their software on the cloud, which means that retinal images are uploaded to the cloud for analysis, with the result then delivered back to the user on the ground. With the cloud hosted outside of Tanzania, and therefore the retinal images also being transferred out of Tanzania during analysis, a cloud-based AI system would breach Tanzanian data privacy laws, irrespective of the level of encryption provided by such processes. Offline capability is not something all AI companies offer as it requires the AI code to be installed locally on a device. Some companies are concerned that this exposes their intellectual property (i.e. their AI software code) to risk if the local device is lost or stolen.

There are no AI systems licenced for DR screening that have been trained using African data and there are ongoing concerns that the under-representation of populations from low-resource settings, such as Tanzania, could mean that AI models perform less well in these populations. To mitigate this, published validation data demonstrating performance within an African population was also an important consideration.

## Selected AI system

After considering all of the above, it was decided that the SELENA+ AI system (owned by EyRis PTE, Singapore) was the most suitable choice. This system has European, South Africa and UAE regulatory approval among others, can function offline and has published validation data from Zambia indicating good performance in a neighbouring African population. The system was installed remotely onto the study laptop in Tanzania, which took 2–3 h. It was important to ensure the laptop had the required hardware to be able to run the AI software efficiently.

The system has an intuitive interface, which after entering some basic demographic information, requires the user to simply drag and drop the JPG retinal image files into the appropriate section of the page. Then, after clicking submit the user receives the result, which states after a few seconds whether the patient is ‘Referable’ or ‘Non-referable’. The Kilimanjaro screening programme medical photographer was trained to use the system in <1 h.

## Implementation into clinical care

Following the preimplementation considerations, particularly the offline capability, our experience has been that for its specific task the AI device has functioned well in the field. Although crucially sensitivity and specificity data are not yet available, they will be reported with the trial results.

However, as the AI system is trained specifically to detect referable DR, it does not refer people that have other potentially blinding conditions (e.g. glaucoma). These cases would typically be detected and trigger a refer outcome if a human grader or ophthalmologist were reviewing the images. In view of the high burden of other ocular conditions in the demographic being screened (mostly people aged >50 y), it is unclear whether the programme can be fully automated, unless the AI software is able to detect other important conditions in addition to DR. As AI models continue to be developed and improved across a range of ocular conditions, integrating AI detection methods for different conditions should be possible in the near future.

The use of an AI-assisted diagnostic tool enables patients to receive a point-of-care result that requires face-to-face counselling. This may be performed by personnel who have not previously delivered patient counselling. In the context of our AI-assisted DR screening programme this has been the case, with the programme’s medical photographer now tasked with informing patients of their results, answering questions and explaining whether or not they require referral to the ophthalmology clinic. This shift in responsibility for delivering clinical information to patients, which is enabled by the AI device, is much less of a focus within clinical AI research, but is of arguably equal importance. Even if an AI device performs its task with near perfect precision, if this information is not articulated to patients clearly and appropriately, it may not improve clinical outcomes. As AI systems move into clinical implementation, particularly diagnostic devices, increased focus on the quality and delivery of patient counselling will become increasingly important. Recent advances in large language models may help this.

## Conclusion

With careful consideration of the local context before implementation, it is feasible to successfully implement a diagnostic AI-enabled medical device into a DR screening programme in a low-resource setting. Within the context of a screening programme, careful evaluation of the chosen AI system to detect other pathologies, beyond the specific condition being screened for, is important. Quality face-to-face counselling of patients, enabled by AI, is critical to the optimal integration of an AI-based diagnostic device.

## Data Availability

Not applicable.
